# B-mode ultrasound and contrast-enhanced ultrasound-based radiomics interpretable analysis for the prediction of macrotrabecular-massive subtype of hepatocellular carcinoma

**DOI:** 10.1186/s13089-025-00452-2

**Published:** 2025-10-17

**Authors:** Dan Lu, Cheng Qin, Li-Fan Wang, Ling-Ling Li, Yu Li, Li-Ping Sun, Hui Shi, Bo-Yang Zhou, Xin Guan, Yao Miao, Hong Han, Jian-Hua Zhou, Hui-Xiong Xu, Chong-Ke Zhao

**Affiliations:** 1 Department of Ultrasound, Zhongshan Hospital, Institute of Ultrasound in Medicine and Engineering, Fudan University, Shanghai, China; 2 School of Future Technology, Shanghai University, Shanghai, China; 3 Department of Ultrasound, State Key Laboratory of Oncology in South China, Sun Yat-sen University Cancer Center, Provincial Clinical Research Center for Cancer, Guangzhou, Guangdong China; 4 Department of Medical Ultrasound, Center of Minimally Invasive Treatment for Tumor, Shanghai Tenth People’s Hospital, School of Medicine, Tongji University, Shanghai, China

**Keywords:** Macrotrabecular-massive hepatocellular carcinoma, Contrast enhanced ultrasound, Radiomics, SHapley additive explanations, Prognosis

## Abstract

**Background:**

This study aimed to develop and validate an interpretable radiomics model using quantitative features from B-mode ultrasound (BMUS) and contrast-enhanced ultrasound (CEUS) for predicting macrotrabecular-massive (MTM) hepatocellular carcinoma (HCC).

**Methods:**

From October 2020 to September 2023, 344 patients (mean age: 58.20 ± 10.70 years; 275 men) with surgically resected HCC were retrospectively enrolled from three medical centers. Radiomics features were extracted from BMUS and CEUS, followed by a multiple-step feature selection process. BMUS_R_ model (based on BMUS radiomics features), BM + CEUS_R_ model (based on BMUS and CEUS radiomics features) and hybrid_R+C_ model (integrated clinical indicators and radiomic features) were established. These radiomics models’ performance was compared with conventional clinic-radiological (C_C+R_) model using area under the receiver operating characteristic curve (AUC). SHapley Additive exPlanations (SHAP) method was used to interpret model performance. The model’s potential for predicting recurrence-free survival (RFS) was further analyzed.

**Results:**

Among ten distinct machine learning classifiers evaluated, the AdaBoost algorithm demonstrated the highest classification performance. The AUCs of the BM + CEUS_R_ model for identifying MTM-HCC were higher than the BMUS_R_ model and the conventional clinic-radiological model in both validation (0.880 vs. 0.720 and 0.658, both *p* < 0.05) and test sets (0.878 vs. 0.605 and 0.594, both *p* < 0.05). No statistical differences were observed between the BM + CEUS_R_ model and the hybrid_R+C_ model in either set (*p* > 0.05). Additionally, the AdaBoost-based BM + CEUS_R_ model showed promising in stratifying early recurrence-free survival, with *p* < 0.001.

**Conclusion:**

The AdaBoost-based BM + CEUS_R_ model shows promise as a tool for preoperatively identifying MTM-HCC and may also be beneficial in predicting prognosis.

**Supplementary Information:**

The online version contains supplementary material available at 10.1186/s13089-025-00452-2.

## Background

Even with recent advancements in the treatment of hepatocellular carcinoma (HCC), high recurrence rates and poor prognosis continue to be pressing concerns [1]. Different HCC subtypes exhibit significant heterogeneity in clinical presentation, radiology findings, H&E morphology, molecular studies, and outcomes [2, 3]. Understanding and characterizing heterogeneity across HCC subtypes is critically important, as it fundamentally informs core dimensions of patient management—spanning diagnostic decision-making, therapeutic strategy formulation, and prognostic evaluation. However, current noninvasive diagnostic methods are still unable to classify hepatocellular carcinoma preoperatively, thus hindering the implementation of precise treatments.

The macrotrabecular-massive (MTM) subtype, recognized by the WHO in 2019, is characterized by a predominantly (> 50%) macrotrabecular growth pattern, accompanied by satellite lesions and vascular invasion, contributing to its highly aggressive nature and poor prognosis in HCC [4, 5]. Research indicates that this subtype serves as a significant predictor of both overall and early recurrence following surgical resection or radiofrequency ablation [6, 7]. Efforts have been devoted to preoperative prediction of MTM-HCC by imaging methods. Several imaging features have been identified to be associated with MTM-HCC, such as larger size [8], intratumor necrosis [9–11], hypo-enhancing components in the arterial phase (AP) [12], and a high frequency of tumor presence in veins [13]. However, evaluating imaging characteristics is prone to interobserver variability, which can lead to discrepancies among radiologists.

Radiomics transforms medical images into mineable high-dimensional datasets by computationally extracting subvisual quantitative features—including morphological, intensity-based, and textural signatures beyond human perceptual limits [14, 15]. Radiomics has demonstrated potential as a quantitative tool for predicting tumor characteristics that are challenging to visually identify or quantify, such as tumor grading and lesion heterogeneity [16–18]. Promising results have been achieved in using radiomics method to distinguish MTM from non-MTM-HCC based on CT and MRI scans [12]. By leveraging its ability to process quantitative image information, machine learning (ML)-based computational approaches have been introduced to enhance the diagnostic accuracy of disease prediction [19]. However, the limited interpretability inherent in ML-based approaches has constrained the clinical translation of radiomics research findings.

As far as we know, the application of ML radiomics to B-mode ultrasound (BMUS) and contrast-enhanced ultrasound (CEUS) for MTM-HCC prediction has not been previously reported. The objective of this research was to evaluate the diagnostic performance of interpretable ML-based radiomics applied to BMUS and CEUS for detecting MTM-HCC, using postoperative histopathology as the gold standard. In addition, the study aimed to further address model interpretability by linking radiomic features to tumor pathology findings.

## Methods

This multi-institutional study was approved by the ethics committee of the institution (No: 2024-203R), and informed consent was obtained.

### Study patients

**Fig. 1:**
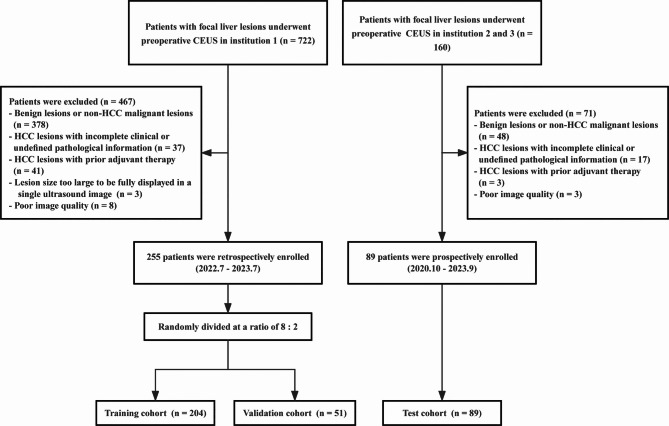
Flowchart of patient selection in this study. CEUS, contrast-enhanced ultrasound; HCC, hepatocellular carcinoma

Data of HCC patients who underwent preoperative B-mode US (BMUS) and CEUS examinations were retrospectively collected at Zhongshan Hospital, Fudan University from July 2022 to July 2023. The inclusion criteria comprised: (a) histopathological confirmation of HCC following surgical resection and (b) preoperative CEUS performed within a 2-week timeframe. The exclusion criteria were as follows: (a) HCC with incomplete clinical information or unclear pathology; (b) previously treated lesions; (c) lesion size too large to be fully displayed in a single US image; (d) poor-quality US data such as incomplete clips for AP, portal venous phase (PVP), or delayed phase (DP); or the lesion could not be recognized on B-mode US. Finally, a total of 255 patients were enrolled in our study following the application of these criteria (Fig. 1).

An external validation set comprising 89 HCC patients was established at two participating institutions (Sun Yat-sen University Cancer Center and Shanghai Tenth People’s Hospital) using the same enrollment criteria applied to the development cohort, with data collected from October 2020 through September 2023.

The electronic medical record system was utilized to record the following clinical indicators: age, sex, infectious status of Hepatitis B virus (HBV) or Hepatitis C virus (HCV), Alpha-Fetoprotein (AFP) level, Albumin (ALB) level, Aspartate Aminotransferase (AST) level, MTM subtype, and postoperative recurrence data. To determine the MTM subtype, pathology slides of the tumor specimens were reviewed by an abdominal pathologist. The MTM subtype is characterized by a predominant (> 50%) architectural pattern (cords of tumor cells thicker than 8 cells) observed upon hematoxylin-eosin staining.

### BMUS and CEUS protocol

All BMUS and CEUS acquisitions were conducted by three board-certified radiologists (each with > 10 years of experience) using standardized ultrasound systems: Samsung RS80A with CA1-7 A transducer (1.0–7.0 MHz), Acuson Sequoia with 5C1 transducer (2.0–5.0 MHz), or GE LOGIQ E9 with C1-5-D transducer (1.0–5.0 MHz) (Supplementary Table 1).

In cases of multiple hepatic tumors, the largest lesion (by diameter) was chosen as the target for assessment. Prior to CEUS examination, BMUS and color Doppler US were initially performed to localize the target lesion. Real-time CEUS imaging was performed on the largest section of the targeted lesion. Dual-screen enabled to simultaneously display the BMUS and CEUS images, with the timer documenting three phases features of CEUS following contrast agent administration. Each patient received either 2.0 mL of SonoVue (Bracco) or 0.6 mL of Sonazoid (GE Healthcare) via manual bolus injection, immediately followed by a 5 mL saline flush (0.9% NaCl). Timer activation was synchronized precisely with contrast bolus injection. The lesion was observed continuously for at least 120 s, and then scanned at 20–30 s intervals and recorded for 5 min or until the microbubbles disappeared. The installed contrast specific imaging mode was coded phase inversion (CPI) with a frame rate of 15–20 fps. CPI is based on pulse inversion harmonic imaging and can enable effective tissue cancellation and avoid destruction of microbubbles in the circulation. All dynamic CEUS cine loops were stored in DICOM format for offline evaluation. Three phases were AP (10–45 s after post-injection), PVP (45–120 s), and DP (121–300 s), respectively.

### Clinic-radiological predictor selection and construction for the conventional clinic-radiological model

Two board-certified radiologists (each with > 3 years of specialized experience in hepatic CEUS) independently reviewed all B-mode ultrasonography and contrast-enhanced cine loops. The readers were blinded to all clinical and pathological data except for the confirmed HCC diagnosis. Any interpretive discrepancies were resolved through consensus discussion between the two radiologists. The following sonographic characteristics were evaluated: a) liver background (cirrhosis/non-cirrhosis); (b) tumor largest diameter; (c) tumor boundary (clear/obscure); (d) halo sign; (e) AP enhancement pattern (homogeneous/heterogeneous); (f) intralesional necrosis (defined as non-enhancing regions persisting during entire CEUS process); (g) PVP enhancement pattern (washout/non-washout).

**Fig. 2:**
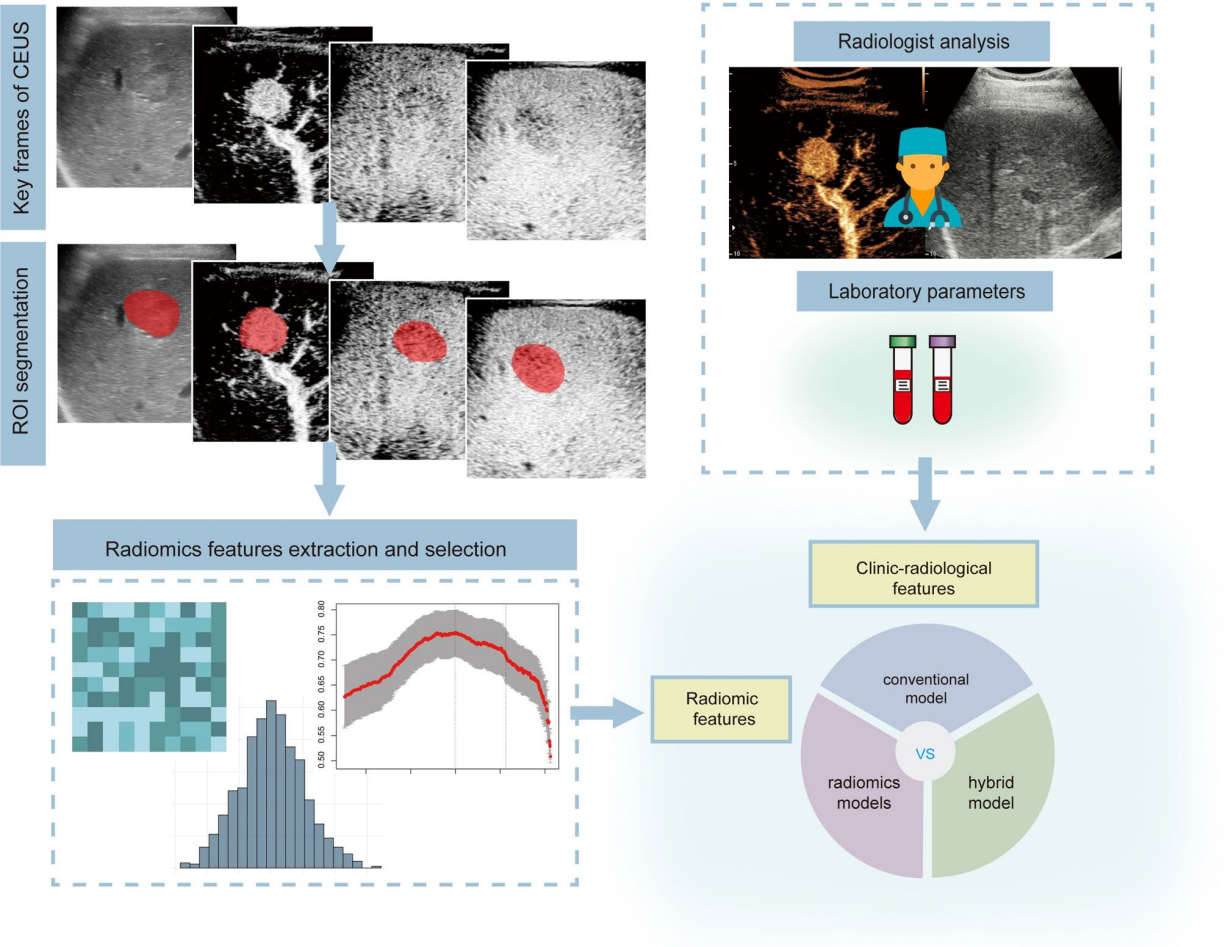
Study workflow of the conventional clinic-radiological model, US radiomics model, and hybrid model

Univariable and multivariable regression analysis was performed on clinical indicators extracted from the institutional electronic medical record system and radiologist interpreted imaging features. The conventional clinic-radiological (C_C+R_) model was developed through multivariable logistic regression analysis, incorporating statistically significant independent predictors identified during model construction. (Fig. 2).

### BMUS and CEUS image segmentation and radiomics features selection

Images of each lesion confirmed by two radiologists (with 7 and 8 years of experience in abdominal US, respectively) who were blinded to the clinicopathologic data in consensus (including 4 US images from BMUS, AP, PVP and DP of CEUS) were used for radiomics analysis. The selection criteria for the three key CEUS images were as follows: for AP, the image showing peak lesion enhancement was selected; for PVP, the image capturing washout (if present) was chosen, otherwise, one image between 90 and 120 s was selected; and for DP, due to intermittent scanning, one image between 180 and 300 s was included.

One radiologist, with three years of experience in abdominal US, manually delineated tumor boundaries by contouring regions of interest (ROIs) using ITK-SNAP software (version 3.6.0; www.itksnap.org). 1070 radiomics features were extracted for each ROI (a total of 4280 features from each patient), using the IFoundry software (Intelligence Foundry 1.2, GE Healthcare) (Supplementary Material S2).

In addition, the same radiologist performed duplicate ROI delineations on 30 randomly selected cases with a 7-day interval between measurements, allowing assessment of segmentation consistency while controlling recall bias. To assess inter-observer variability, an additional radiologist (4 years of specialized US experience) independently contoured ROIs on the same image set, enabling comparative analysis of segmentation consistency between operators. Interclass correlation coefficient (ICC) was used to evaluate the intra- and inter-operator agreement of feature extraction. An ICC > 0.80 was considered excellent.

Radiomics feature selection was performed through a multi-step approach: (1) ICC analysis retaining features with excellent reproducibility (ICC > 0.8); (2) independent samples t-test identifying features with significant discriminative power (*p* < 0.05); (3) Spearman’s rank correlation eliminating redundant features (*r* ≥ 0.8); and (4) LASSO regression with 10-fold cross-validation for final feature selection.

### Construction and validation of the ML-based radiomics models

Ten distinct machine learning algorithms were employed to develop predictive models (Supplementary Material S3) [20]. Selected radiomics features and clinical indicators served as inputs for training different radiomics models. Model optimization was performed via five-fold cross-validation, with subsequent performance evaluation based on the area under the receiver operating characteristic curve (AUC). The algorithm demonstrating superior discriminative ability (highest AUC) in the validation cohort was selected as the optimal radiomics model.

Three type radiomics models were constructed as follows: the ML-based BMUS_R_ model based on BMUS radiomics features; the ML-based BM + CEUS_R_ model based on BMUS and CEUS radiomics features; and the ML-based hybrid_R+C_ model based on BMUS and CEUS radiomics features together with clinical indicators (Fig. 2).

#### Interpretability of the optimal ML model’s performance

ML-based models have interpretability issues because they work like a “black box”. In this study, the SHAP algorithm was employed to explain the prediction results of the best-performing model using Python open-source SHAP package. SHAP algorithm is based on the Shapley value theory, decomposing the prediction results into the impact of each feature, providing interpretability for the model [21–23]. The summary plot provides insights into both feature importance and feature effects. Each point on the plot corresponds to a SHAP value for a specific feature and instance. It illustrates the relationship between feature values and their influence on the diagnosis. The heatmap reflects the specific impact of each feature on each sample.

### The relationship between the MTM subtype and postoperative recurrence

The primary outcome measure was recurrence-free survival (RFS), calculated from the date of curative resection to the first documented event of tumor recurrence (either intrahepatic or extrahepatic) or mortality. Intrahepatic recurrence was characterized by the emergence of new tumors within the liver. Patients were followed until recurrence, death, or the end date of this study. Follow-up data was gathered and reviewed from patients’ medical records. Comprehensive follow-up data were obtained through systematic medical record review supplemented by structured telephone interviews for patients lost to clinical follow-up.

### Statistical analysis

All statistical analyses were conducted using Python 3.8.8 and SPSS Statistics 22.0. Normally distributed continuous variables were expressed as mean ± standard deviation and compared using independent samples t-tests. Categorical variables were presented as frequencies and analyzed with Pearson’s chi-square test. Diagnostic accuracy was assessed by calculating the area under the receiver operating characteristic curve (AUC) with 95% confidence intervals. Univariate and multivariate analyses were performed to select the clinical indicators. DeLong’s test was used to assess differences between AUCs. The RFS rates were calculated using the Kaplan-Meier estimator, with between-group comparisons performed via log-rank testing. A *p-*value of less than 0.05 was considered statistically significant.

## Result

### Baseline characteristics

Eventually, a total of 255 HCC patients (202 men and 53 women) fulfilled eligibility criteria were included for model construction. They were randomly assigned to the training and validation sets in an 8-to-2 ratio. 65 HCC patients from center 1 and 24 HCC patients from center 2, respectively, constituted the test set. The clinic-radiological characteristics of patients in the training, internal validation, and external test sets are summarized in Table 1.

**Table 1:** The clinic-radiological characteristics of HCC patients in the training set, internal validation set, and external test set

Characteristics	Total	Training set	Validation set	Test set
No. of patients	344	204	51	89
Sex				
Female	69 (20.06)	43 (21.08)	10 (19.61)	16 (17.98)
Male	275 (79.94)	161 (78.92)	41 (80.39)	73 (82.02)
Mean age, year	58.20 ± 10.70	58.79 ± 10.16	59.90 ± 11.02	55.88 ± 11.46
(range)*	(25, 87)	(25, 87)	(29, 79)	(26, 77)
MTM subtype	103 (29.94)	71 (34.80)	15 (29.41)	17 (19.10)
HBV/HCV history	298 (86.63)	176 (86.24)	43 (84.31)	79 (88.76)
AFP				
≤ 40 ng/mL	206 (59.88)	119 (58.33)	31 (60.78)	56 (62.92)
> 40 ng/mL	138 (40.12)	85 (41.67)	20 (39.22)	33 (37.08)
ALB				
< 29 g/L	16 (4.65)	9 (4.41)	4 (7.84)	3 (3.37)
≥ 29 g/L	328 (95.35)	195 (95.59)	47 (92.16)	86 (96.63)
Mean tumor size, cm	4.24 ± 2.43	4.00 ± 2.69	3.98 ± 2.25	4.93 ± 1.70
(range)*	(0.8, 13)	(0.8, 13)	(1, 11)	(1, 10.9)
Cirrhosis	146 (42.44)	96 (47.06)	19 (37.25)	31 (34.83)
Margin				
Clear	67 (19.48)	41 (20.00)	10 (19.61)	16 (17.98)
Obscure	277 (80.52)	163 (80.00)	41 (80.39)	73 (82.02)
Halo sign	61 (17.73)	38 (18.63)	9 (17.65)	14 (15.73)
AP heterogeneous enhancement	283 (82.27)	74 (36.27)	18 (35.29)	32 (35.96)
PVP hypo-enhancement	114 (33.14)	69 (33.82)	17 (33.33)	28 (31.46)
Necrosis	36 (10.47)	27 (13.24)	3 (5.88)	6 (6.74)

Data in parentheses are percentages except for special indications

*MTM*, macrotrabecular-massive; *HBV*, Hepatitis B Virus; *HCV*, Hepatitis C Virus; *AFP*, Alpha-fetoprotein; *ALB*, Albumin; *AP*, arterial phase; *PVP*, portal venous phase

*Data are presented as the mean ± standard deviation, with ranges in parentheses

### Construction and validation of the C_C+R_ model and the ML-based US radiomics models

**Fig. 3:**
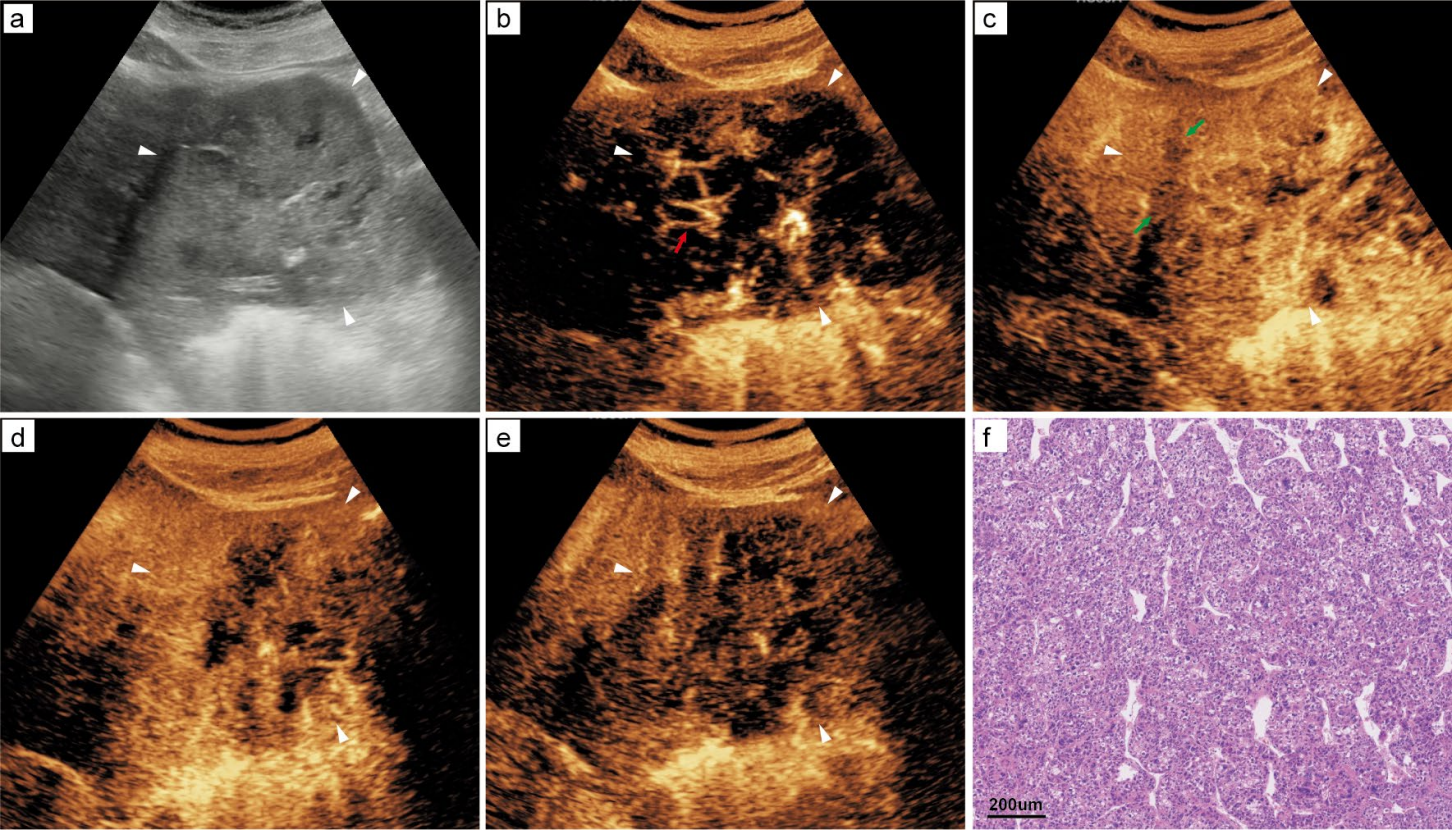
A case of MTM-HCC in a 57-year-old male participant with hepatitis B virus–related cirrhosis.** a**–**e** Images from preoperative CEUS demonstrate an 9.4-cm HCC in the left anterior lobe of the liver; the lesion is marked by white arrowheads.** a** The lesion exhibited an ill-defined margin with heterogeneous echotexture.** b**,** c** A visible intratumoral artery (red arrow) and hypoenhancing component (green arrow) during the arterial phase.** d** Early heterogeneous washout was observed at 55 s.** e** Delayed phase image showed marked washout extending beyond the margins of the B-mode lesion.** f** Photomicrograph reveals a macrotrabecular pattern. (Hematoxylin-eosin stain)

After univariate and multivariate logistic regression analysis, the cirrhosis, PVP hypo-enhancement, elevated AFP level (> 40 ng/mL), and lowered albumin level (< 29 g/L) were decided as independent risk factors of MTM-HCC in the training set (all *p* < 0.05) (Table 2). CEUS and histopathological images of a representative MTM-HCC case (Fig. 3). For the prediction of MTM-HCC, the C_C+R_ model achieved an AUC of 0.658, a sensitivity of 72.2%, a specificity of 54.5%, a PPV of 46.4%, a NPV of 78.3%, and an accuracy of 60.8% in the validation set (Table 4).

**Table 2:** The univariate and multivariate logistic regression analysis in clinic-radiological features for the prediction of MTM-HCC

Characteristics	Univariate analysis		Multivariate analysis	
	OR (95% CI)	*p* value	OR (95% CI)	*p* value
HBV/HCV history				
Absence vs. presence	0.615 (0.275,1.373)	0.235	/	/
AFP				
≤ 40 vs. > 40 ng/mL	0.374 (0.219, 0.638)	< 0.001	0.433 (0.246, 0.761)	0.004
ALB				
≥ 29 vs. < 29 g/L	0.137 (0.037, 0.513)	0.003	0.147 (0.037, 0.579)	0.006
Tumor size	1.154 (1.043, 1.276)	0.005	1.083 (0.952, 1.233)	0.227
Cirrhosis				
Absence vs. presence	0.585 (0.285, 0.824)	0.007	0.492 (0.279, 0.871)	0.015
Margin				
Clear vs. obscure	0.541 (0.267, 1.096)	0.088	/	/
Halo sign				
Absence vs. presence	0.783 (0.406, 1.507)	0.464	/	/
AP enhancement pattern				
Homogeneous vs. heterogeneous	0.474 (0.278, 0.811)	0.006	0.742 (0.380, 1.449)	0.382
PVP hypo-enhancement				
Absence vs. presence	0.430 (0.238, 0.783)	0.006	0.455 (0.244, 0.851)	0.014
Necrosis				
Absence vs. presence	0.263 (0.118, 0.587)	0.001	0.464 (0.173, 1.243)	0.127

*HBV*, Hepatitis B Virus; *HCV*, Hepatitis C Virus; *AFP*, Alpha-fetoprotein; *ALB*, Albumin; *AP*, arterial phase; *PVP*, portal venous phase

**Table 3:** The AUC results of ten ML algorithms for the BMUS_R_, BM + CEUS_R,_ and hybrid_R+C_ models

ML algorithms	BMUS_*R*_		BM + CEUS_*R*_		Hybrid_*R*+C_	
	AUC (95% CI)	*p* value	AUC (95% CI)	*p* value	AUC (95% CI)	*p* value
AdaBoost	0.720 (0.554,0.885)	/	0.880 (0.820, 0.935)	/	0.914 (0.813, 1.000)	/
Gradient boosting	0.704 (0.542,0.865)	0.865	0.794 (0.632, 0.937)	0.113	0.808 (0.687, 0.929)	0.190
XGBoost	0.680 (0.508,0.802)	0.709	0.836 (0.671, 0.955)	0.212	0.841 (0.721, 0.955)	0.348
Bagging	0.649 (0.488,0.811)	0.649	0.767 (0.528, 0.960)	0.166	0.814 (0.678, 0.951)	0.252
Decision tree	0.554 (0.389,0.720)	0.127	0.614 (0.464, 0.761)	< 0.001	0.678 (0.545, 0.812)	0.007
Extra trees	0.712 (0.555,0.869)	0.903	0.666 (0.487, 0.830)	0.007	0.775 (0.629, 0.920)	0.127
Logistics regression	0.616 (0.449,0.784)	0.381	0.666 (0.498, 0.819)	0.003	0.733 (0.596, 0.871)	0.041
Naïve Bayes	0.701 (0.541,0.860)	0.828	0.783 (0.652, 0.914)	0.362	0.733 (0.596, 0.871)	0.052
Random forest	0.607 (0.436,0.779)	0.338	0.790 (0.637, 0.915)	0.173	0.721 (0.555, 0.875)	0.052
K-nearest neighbour	0.563 (0.322,0.803)	0.286	0.621 (0.462, 0.789)	< 0.001	0.653 (0.505, 0.800)	0.005

C_C+R_ model, based on clinic-radiological characteristics; BMUS_R_ model, based on B-mode US radiomics features; BM + CEUS_R_ model, based on B-mode US and CEUS radiomics features; Hybrid_c+R_ model, based on clinical features and B-mode US and CEUS radiomics features;

ML, machine learning. AUC, area under the curve. CI, confidence interval

**Table 4:** The diagnostic performance of various models in discrimination MTM from non-MTM ones

Models	AUC	SEN	SPE	PPV	NPV	ACC	*p* value*
C_C+*R*_ model							
Validation set	0.658 (0.499, 0.817)	72.2 (51.5, 92.9)	54.5 (37.6, 71.5)	46.4 (28.0, 64.9)	78.3 (61.4, 95.1)	60.8 (59.9, 61.7)	0.029
Test set	0.594 (0.449, 0.740)	64.7 (42.0, 87.4)	50.0 (38.5, 61.5)	23.4 (11.3, 35.5)	0.857 (75.1, 96.3)	0.528 (52.3, 53.4)	0.005
BMUS_R_ model							
Validation set	0.720 (0.554, 0.885)	58.8 (35.4, 82.2)	88.2 (77.4, 99.1)	71.4 (47.8, 95.1)	81.1 (68.5, 93.7)	78.4 (77.8, 79.1)	0.121
Test set	0.605 (0.478, 0.731)	88.2 (72.9, 100)	43.1 (31.6, 54.5)	26.8 (15.2, 38.4)	93.9 (85.8, 102)	51.7 (51.1, 52.2)	0.012
BM + CEUS_R_ model							
Validation set	0.880 (0.766, 0.995)	77.8 (58.6, 97.0)	90.9 (81.1, 100)	82.4 (64.2, 100)	88.2 (77.4, 99.1)	86.3 (85.8, 86.7)	/
Test set	0.878 (0.769, 0.987)	82.4 (64.2, 100)	88.9 (81.6, 96.1)	63.6 (43.5, 83.7)	95.5 (90.6, 100)	87.6 (87.4, 87.9)	/
Hybrid_R+C_ model							
Validation set	0.914 (0.813, 1.00)	80.0 (62.5, 97.5)	100 (100, 100)	100 (100, 100)	89.7 (80.2, 99.3)	92.7 (92.5, 93.0)	0.574
Test set	0.892 (0.776, 1.00)	82.4 (64.2, 100)	93.1 (87.2, 98.9)	73.7 (53.9, 93.5)	95.7 (91.0, 100)	91.0 (90.8, 91.2)	0.112

Data in parentheses are 95% confidence interval

C_C+R_ model, based on clinic-radiological characteristics; BMUS_R_ model, based on B-mode US radiomics features; BM + CEUS_R_ model, based on B-mode US and CEUS radiomics features; hybrid_R+C_ model, based on clinical features and B-mode US and CEUS radiomics features;

*SEN*, sensitivity; *SPE*, specificity; *PPV*, positive predictive value; *NPV*, negative predictive value; *AUC*, area under the curve. * *P* value of AUCs differ from BM + CEUS_R_ model

The feature selection process employed a multi-step approach: after ICC analysis, features were reduced from 4,280 to 3,437; following t-test, features decreased to 914; after Spearman’s rank correlation, features were further reduced to 666; and finally, LASSO regression narrowed features down to 47. These 47 radiomics features included 11 radiomics features from BMUS, 11 radiomics features from AP of CEUS, 11 radiomics features from PVP of CEUS, and 14 radiomics features from DP of CEUS (Supplementary Fig. 4 and Supplementary Table 5).

For the BMUS_R_ model, the AdaBoost classifier with the selected 11 radiomics features showed the highest AUC of 0.720 compared with other 9 ML classifiers (AUC: 0.554–0.712) in the validation set for discriminating between MTM and non-MTM HCC lesions. The sensitivity, specificity, PPV, NPV, and accuracy of the model were 58.8%, 88.2%, 71.4%, 81.1%, and 78.4%, respectively (Tables 3 and 4).

For the BM + CEUS_R_ model, the AdaBoost algorithm with the selected 47 radiomics features showed the highest AUC of 0.880 in comparison to other 9 ML algorithms (AUC: 0.614–0.836) in the validation set for discriminating between MTM and non-MTM HCC lesions. The training curve of BM + CEUS_R_ model was shown in Supplementary Fig. 6. The sensitivity, specificity, PPV, NPV, and accuracy of the model were 77.8%, 90.9%, 82.4%, 88.2%, and 86.3%, respectively (Tables 3 and 4).

For the hybrid_R+C_ model, the AdaBoost algorithm with 47 radiomic features and two laboratory features showed the highest AUC of 0.914 compared with other 9 ML algorithms (AUC: 0.653–0.841) in the validation set for discriminating between MTM and non-MTM HCC lesions. The sensitivity, specificity, PPV, NPV, and accuracy of the model were 80.0%, 100%, 100%, 89.7%, and 92.7%, respectively (Tables 3 and 4).

### Comparison between the C_C+R_ model and three types of radiomics models

**Fig. 4:**
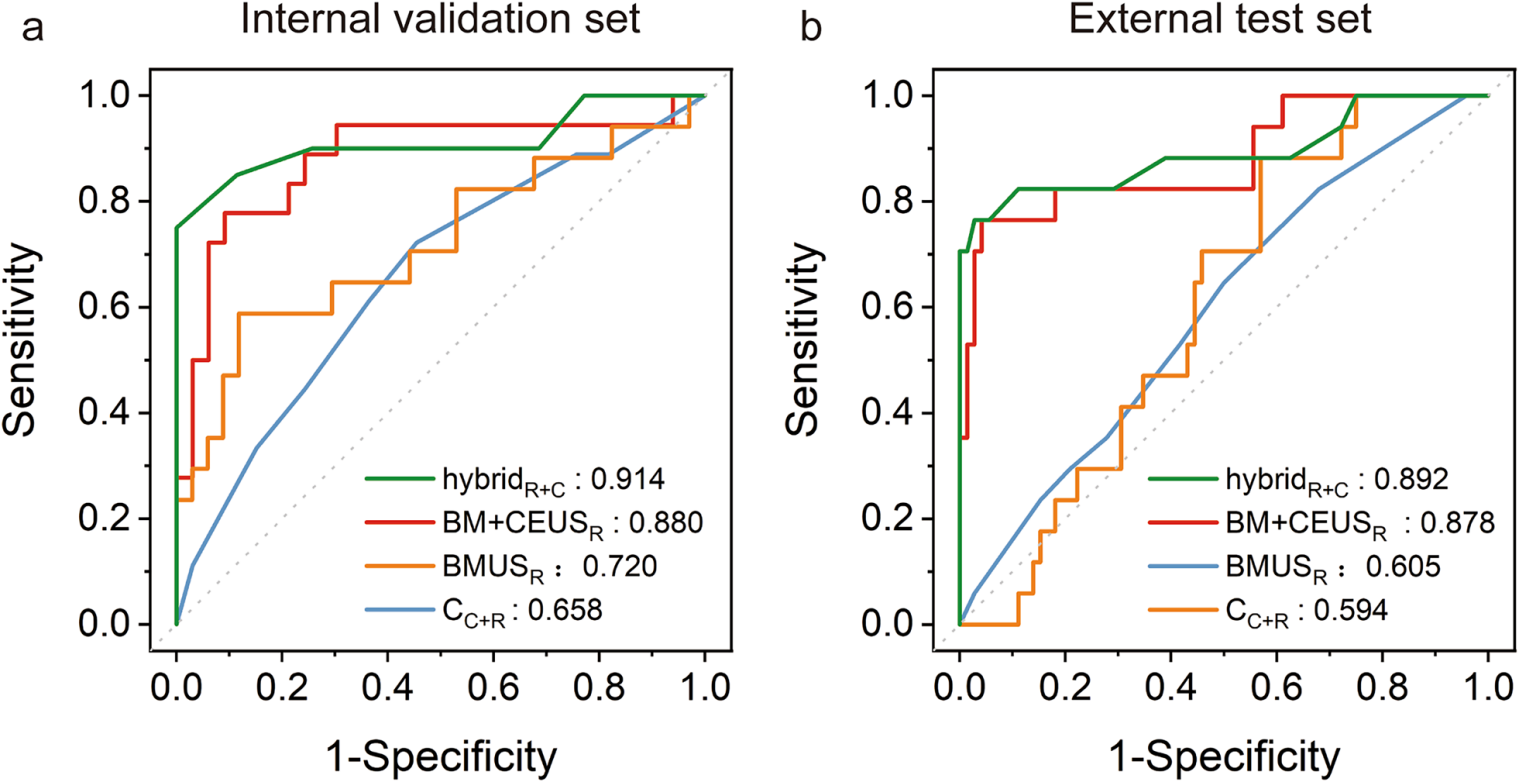
Receiver operating characteristic (ROC) curves of the C model, BMUS_R_ model, BM + CEUS_R_ model, and hybrid_R+C_ model in the internal validation (**a**) and external test (**b**) sets. The conventional clinic-radiological (C_C+R_) model, based on clinic-radiological characteristics; The BMUS_R_ model, based on B-mode US radiomics features; The BM + CEUS_R_ model, based on B-mode and CEUS radiomics features; The hybrid_R+C_ model, based on CEUS radiomics features and clinic-radiological features

The diagnostic results of the C_C+R_ model and three types of radiomics models in internal validation and external test sets were shown in Table 3; Fig. 4.

The AdaBoost-based BMUS_R_ model showed a similar performance compared to that of the C_C+R_ model in terms of AUCs (0.720 vs. 0.658 and 0.605 vs. 0.594, both *p* > 0.05) in the internal validation and external test set, respectively.

The AdaBoost-based BM + CEUS_R_ model showed a better performance compared to that of the C_C+R_ model in terms of AUCs (0.880 vs. 0.658 and 0.878 vs. 0.594, both *p* < 0.05) in the internal validation and external test set, respectively. In comparison of the AdaBoost-based BM + CEUS_R_ model and the AdaBoost-based BMUS_R_ model, no statistical difference was found between the two models in terms of AUCs (0.880 vs. 0.720, *p* > 0.05) in the internal validation set. However, in the external test set AdaBoost-based BM + CEUS_R_ model demonstrated superior performance compared to the AdaBoost-based BMUS_R_ model in terms of AUCs (0.878 vs.0.605, *p* < 0.05).

No statistical difference was found between the AdaBoost-based hybrid_R+C_ model and the AdaBoost-based BM + CEUS_R_ model in the internal validation and external test set (0.914 vs. 0.880 and 0.892 vs. 0.878, both *p* > 0.05).

### The ML-based model interpretation

**Fig. 5:**
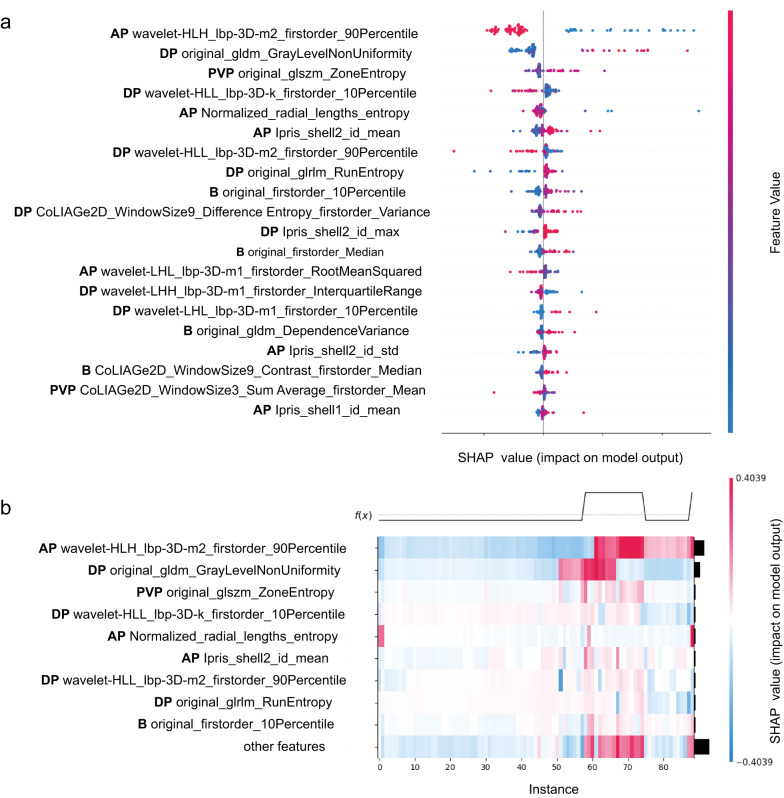
SHAP interpretation of the BM + CEUS_R_ model.** a** The top 20 radiomics features ranked by importance, evaluated with stability and interpretability using the CEUS_R_ model. The higher SHAP value of a feature is given, the higher risk of MTM-HCC would be. The red section in the feature value indicates a higher value.** b** The heatmap reflects the specific impact of a single feature on each sample in the external test set. *AP* arterial phase, *DP* delayed phase, *PVP* portal venous phase, *B* b-mode

SHAP values represent the impact of each feature on the final prediction, providing a clear and effective explanation of model predictions for individual patients. The SHAP beeswarm plot showed the top 20 crucial radiomics features in BM + CUES_R_ model (Fig. 5a). The blue and red dots indicate whether a factor decreased (blue) or increased (red) the risk of MTM subtype. The SHAP heatmap illustrates the impact of the top 9 radiomics features on each individual case in the external set, with darker colors indicating a greater impact (Fig. 5b).

### The relationship between the MTM subtype and postoperative recurrence

**Fig. 6:**
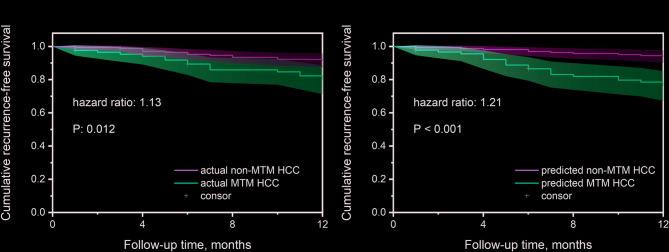
The recurrence-free survival rates in actual non-MTM patients and MTM patients** a** and BM + CEUS_R_ model predicted non-MTM patients and MTM patients** b** in center 1

For the 255 patients in center 1, the median follow-up period was 16.15 months (interquartile range, 13–25 months). Three patients were lost to follow-up within one year, one of whom was MTM positive. The recurrence-free survival rates at 3, 6, and 12 months for the whole study population were 97.23%, 92.86%, and 88.10%, respectively. 14 (8.38%) in non-MTM patients and 16 (19.05%) in MTM patients experienced intrahepatic recurrences within one year after surgery (Fig. 6a). There was a statistically significant difference in the one-year postoperative recurrence rates between non-MTM and MTM-HCC as shown in stratification analysis of Kaplan-Meier curves of RFS. For the BM + CEUS_R_ model, significant differences in RFS were seen between the predicted MTM-HCC and non-MTM-HCC patients (log-rank test, *p* < 0.001, Fig. 6b).

For the 89 patients from center 2 and 3, the median follow-up period was 23.76 months (interquartile range, 1–47 months). A total of 16 patients were lost to follow-up within one year. The one-year recurrence-free survival rates were 92.71% and 91.67% in non-MTM patients and MTM patients, respectively. No statistically significant difference in one-year postoperative recurrence rates was found in either actual non-MTM patients and MTM patients or model predicted non-MTM patients and MTM patients (Supplementary Fig. 7).

## Discussion

In this study, we built and validated an interpretable ML model to identify high risk MTM-HCC from the other HCC subtypes. The AUCs of the BM + CEUS_R_ model for discriminating MTM-HCC from non-MTM HCC were higher than the BMUS_R_ model and the C_C+R_ model in the external test set (0.878 vs. 0.605 and 0.594, *p* < 0.05). The BM + CEUS_R_ and hybrid_R+C_ models demonstrated comparable performance in both test sets (*p* > 0.05), suggesting similar discriminative capability regardless of feature composition. Additionally, the AdaBoost-based BM+CEUS_R_ model demonstrated significant prognostic power in stratifying early recurrence-free survival (less than 1 year), with *p* < 0. 001.The model also exhibited promising interpretability, allowing for a better understanding of the factors contributing to its predictions.

MTM subtype is a significant risk factor for tumor recurrence following surgery in HCC and can only be confirmed through microscopic examination. Identifying MTM-HCC before treatment can offer valuable prognostic insights and lead to the implementation of a more rigorous follow-up strategy. Many studies have focused on the identification of MTM-HCC due to its poor prognosis in recent years. Previous investigations utilizing conventional imaging biomarkers and clinical variables for MTM-HCC prediction have demonstrated variable diagnostic performance, with reported AUC from 0.69 to 0.89 [8, 9, 11; 24]. In the past two years, an ever-growing number of studies have focused on radiomics analysis in MTM subtype prediction based on CT or MRI [25–28]. Li et al. and Feng et al. constructed CT-based radiomics models for MTM subtype prediction and achieved the AUC of 0.89 and 0.74, respectively [25, 26]. For MRI-related study, Zhang et al. developed a multiparametric MRI radiomics model incorporating both imaging signatures and clinic-radiological variables, reporting discriminative performance of 0.81 [28]. As far as we know, this study was the first to perform ML analysis to explore the performance of CUES-based radiomics in prediction of MTM-HCC preoperatively.

As a supplement to conventional US, CEUS can significantly improve the diagnostic efficiency for focal liver lesions and is recommended as a routine examination for HCC at-risk patients in the guidelines [29, 30]. For the construction of ML models, radiomics features from BMUS images and key frames at different phases of CEUS were employed. ML model based on BMUS and CEUS radiomics features (BM + CEUS_R_ model) showed superior predictive performance than ML model based on BMUS radiomics features (BMUS_R_ model). MTM-HCC has been reported to be linked to a distinctive microvascular pattern, referred to as a sinusoid-like microvascular pattern characterized by a cobweb-like network of micro-vessels that surround individual HCC clusters [31]. HCCs exhibiting this vascular pattern are associated with low microvascular density and a high incidence of tumor necrosis [32, 33]. This explains why the performance of BM + CEUS_R_ model is significantly better than that of the BMUS_R_ model, as it can reflect valuable information about tumor perfusion in addition to morphological features.

For the construction of the conventional clinic-radiological model, except for the CEUS imaging features obtained from human interpretation, laboratory indicators were also evaluated. Independent factors included cirrhosis, PVP hypo-enhancement, elevated AFP level (> 40 ng/mL), and lowered albumin level (< 29 g/L) which was similar with that in other studies [7, 24]. However, the performance of C_C+R_ model was far from satisfactory with an AUC of 0.658 and 0.594 in the internal and external test set respectively. What’s more, the inclusion of clinical indicators failed to improve the BM + CEUS_R_ model’s performance in either the internal or the external test set (both *p* > 0.05). We consider that substantial information overlaps between clinical features and radiomics features is the main reason for the limited contribution of clinical variables. Multiple papers confirm that radiomics features and serum biomarkers often measure different manifestations of the same underlying biological processes. Rizzo et al. explicitly mentioned the correlation between certain radiomic features and serum CA-125 level in ovarian cancer [34]. Ji et al. found associations between CT arterial phase texture and serum AFP, reflecting shared biological information about tumor aggressiveness and behavior [35]. Besides, we consider that imaging features interpreted by radiologists are inherently subjective and macroscopic which may have limited value in predicting MTM subtype. In contrast to this, the texture features included in radiomics are at the microscopic scale and can be objectively quantified and calculated. Texture analysis focuses on the most fundamental informational features within an image which enables quantification of image heterogeneity caused by changes imperceptible to the human eye [36]. Research indicated a strong correlation between radiomic features and cellular-level heterogeneity indices [37, 38]. Radiomics offers potential as a complementary tool for non-invasive tumor characterization and quantification of intra-tumoral heterogeneity [39–41]. As the pathological diagnostic criterion of MTM subtype, predominant (i.e., >50% of the tumor area) macrotrabecular architecture is a change at the histocytological level. BM + CEUS_R_ model based on radiomics features can effectively capture subtle changes in BMUS and CEUS images, thus demonstrating outstanding model performance compared to the C_C+R_ model.

From the interpretability SHAP plots of the BM + CEUS_R_ model, we can see that the top-ranked radiomic features mainly acquired from the arterial phase and the delayed phase (Fig. 4a and b). MTM-HCC is reported to be associated with LR-M features, particularly the rim-like enhancement with central hypoenhancing areas in the arterial phase [13, 42]. What’s more, AP hypovascular component has been reported as an important characteristic of MTM-HCC in many studies on CT and MRI [12, 43, 44]. In some other studies, MTM-HCC exhibited a poorly differentiated status [7, 45], which would show higher washout rate [46, 47]. The above points explain why radiomics features from AP and DP carry more weight in the BM + CEUS_R_ model.

In addition to the detection of MTM subtype, the model’s potential in prognosis prediction is also a point of interest for us. Patients with pathologically confirmed MTM-HCC in center 1 exhibited a higher recurrence rate within one year after surgery, which is consistent with previous studies [7]. The survival curve of MTM-HCC and non-MTM-HCC predicted by the BM + CEUS_R_ model was close to the actual RFS survival time. A statistical difference in 1-year RFS was shown between the predicted MTM-HCC and non-MTM-HCC patients in center 1 which indicated that the BM + CEUS_R_ model might be promising in stratifying the prognosis of HCC patients. The limited sample size may account for the lack of statistically significant differences in 1-year RFS observed in the external centers.

This study has several limitations. First, due to the relatively low proportion of the MTM subtype in HCC, the sample size for this subtype is relatively small when constructing predictive models, which may lead to bias in ML. Second, the dataset was predominantly composed of patients of East Asian descent, with a high incidence of HBV-related HCC. Consequently, caution is essential when applying the model’s findings to HCC patients without history of HBV. Additional validation in varied patient populations is necessary in the future. Third, the retrospective nature of the study introduces inherent biases. To improve the reliability of the results, future research should include larger, well-structured prospective datasets for both model training and validation.

In summary, our study introduced an ML-assisted radiomics model based on BMUS and CEUS for precise prediction of MTM subtype and RFS in patients with HCC.

## Supplementary Information

Below is the link to the electronic supplementary material.

Supplementary Material 1.

Supplementary Material 2.

Supplementary Material 3.

Supplementary Material 4.

Supplementary Material 5.

Supplementary Material 6.

Supplementary Material 7.

## Data Availability

The datasets used or analyzed during the current study are available from the corresponding author on reasonable request.
