# Correction: B-mode ultrasound and contrast-enhanced ultrasound-based radiomics interpretable analysis for the prediction of macrotrabecular-massive subtype of hepatocellular carcinoma

**DOI:** 10.1186/s13089-025-00468-8

**Published:** 2025-11-25

**Authors:** Dan Lu, Cheng Qin, Li-Fan Wang, Ling-Ling Li, Yu Li, Li-Ping Sun, Hui Shi, Bo-Yang Zhou, Xin Guan, Yao Miao, Hong Han, Jian-Hua Zhou, Hui-Xiong Xu, Chong-Ke Zhao

**Affiliations:** 1 Department of Ultrasound, Zhongshan Hospital, Institute of Ultrasound in Medicine and Engineering, Fudan University, Shanghai, China; 2 School of Future Technology, Shanghai University, Shanghai, China; 3 Department of Ultrasound, State Key Laboratory of Oncology in South China, Sun Yat-sen University Cancer Center, Provincial Clinical Research Center for Cancer, Guangzhou, Guangdong, China; 4 Department of Medical Ultrasound, Center of Minimally Invasive Treatment for Tumor, Shanghai Tenth People’s Hospital, School of Medicine, Tongji University, Shanghai, China

**Correction: The Ultrasound Journal (2025) 17: 53**, 10.1186/s13089-025-00452-2.

Following publication of the original article [1], it was noted that Figs. 3 and 4 were wrongly numbered. Figure 1 should have been Fig. 3 and Fig. 3 should have been Fig. 4. In the original article, Fig. 1 was missing, and Fig. 6 was duplicated as Fig. 4.

All figure captions were correct in the original article and have not been adjusted.

The original article [1] has been corrected.
