# Supplementary material for: B-mode ultrasound and contrast-enhanced ultrasound-based radiomics interpretable analysis for the prediction of macrotrabecular-massive subtype of hepatocellular carcinoma
Source: Ultrasound J. 2025 Oct 17;17:53. doi: 10.1186/s13089-025-00452-2 (PMC12534629; doi:10.1186/s13089-025-00452-2)
Supplement: Supplementary file 1 — Supplementary Material 1. [file 13089_2025_452_MOESM1_ESM.docx]

S2. The extracted US radiomics features details

The 1070 ultrasonographic radiomics features comprised 5 contour features, 8 length-based measurements, 13 shape descriptors, 13 textural phenotype parameters, and 23 gray-level co-occurrence matrix (GLCM) features. Additionally included were 16 gray-level run-length matrix (GLRLM) features, 16 gray-level size-zone matrix (GLSZM) features, 5 neighborhood gray-tone difference matrix (NGTDM) features, 13 gray-level dependence matrix (GLDM) features, 48 intra-perinodular textural transition (Ipris) features, 442 wavelet-transform local binary pattern (LBP) features, and 468 co-occurrence of local anisotropic gradient orientations (CoLIAGe) features.
