# Supplementary material for: B-mode ultrasound and contrast-enhanced ultrasound-based radiomics interpretable analysis for the prediction of macrotrabecular-massive subtype of hepatocellular carcinoma
Source: Ultrasound J. 2025 Oct 17;17:53. doi: 10.1186/s13089-025-00452-2 (PMC12534629; doi:10.1186/s13089-025-00452-2)
Supplement: Supplementary file 2 — Supplementary Material 2. [file 13089_2025_452_MOESM2_ESM.docx]

S3. The ten ML classifiers used to construct ML-based US radiomics models

**AdaBoost**
A boosting technique that assigns equal weights to all training samples initially and iteratively adjusts these weights by focusing more on misclassified datapoints for next model.

**Gradient Boosting**
A boosting algorithm which combine multiple weak learner to create a strong predictive model.

**XGBoost**
A type of ensemble learning method that combines multiple weak models to form a stronger model.

**Bagging**
A type of ensemble learning in which multiple base models are trained independently and parallelly on different subsets of training data. The final prediction is made by aggregating the predictions of all base model using majority voting.

**Decision Tree**
A classifier has a tree-like structure that starts with one main question called the root node which represents the entire dataset. Tree branches out into different possibilities based on features in the data.

**Extra Trees**
A type of ensemble learning technique which aggregates the results of multiple de-correlated decision trees collected in a "forest" to output it's classification result.

**Random Forest**
A machine learning algorithm that uses many decision trees to make better predictions. Each tree looks at different random parts of the data and their results are combined by voting for classification or averaging for regression.

**Logistic Regression**
An machine learning approach uses the sigmoid function to simulate the likelihood of an instance falling into a specific class, producing values between 0 and 1.

**Naïve Bayes**
A classification algorithm that uses probability to predict which category a data point belongs to, assuming that all features are unrelated.

**K-Nearest Neighbour**
A supervised machine learning algorithm works by finding the "k" closest data points (neighbors) to a given input and makesa predictions based on the majority class (for classification) or the average value (for regression).
