# Supplementary material for: B-mode ultrasound and contrast-enhanced ultrasound-based radiomics interpretable analysis for the prediction of macrotrabecular-massive subtype of hepatocellular carcinoma
Source: Ultrasound J. 2025 Oct 17;17:53. doi: 10.1186/s13089-025-00452-2 (PMC12534629; doi:10.1186/s13089-025-00452-2)
Supplement: Supplementary file 3 — Supplementary Material 3. [file 13089_2025_452_MOESM3_ESM.docx]

Figure S4. Results of feature reduction using the least absolute shrinkage and selection operator (LASSO) regression


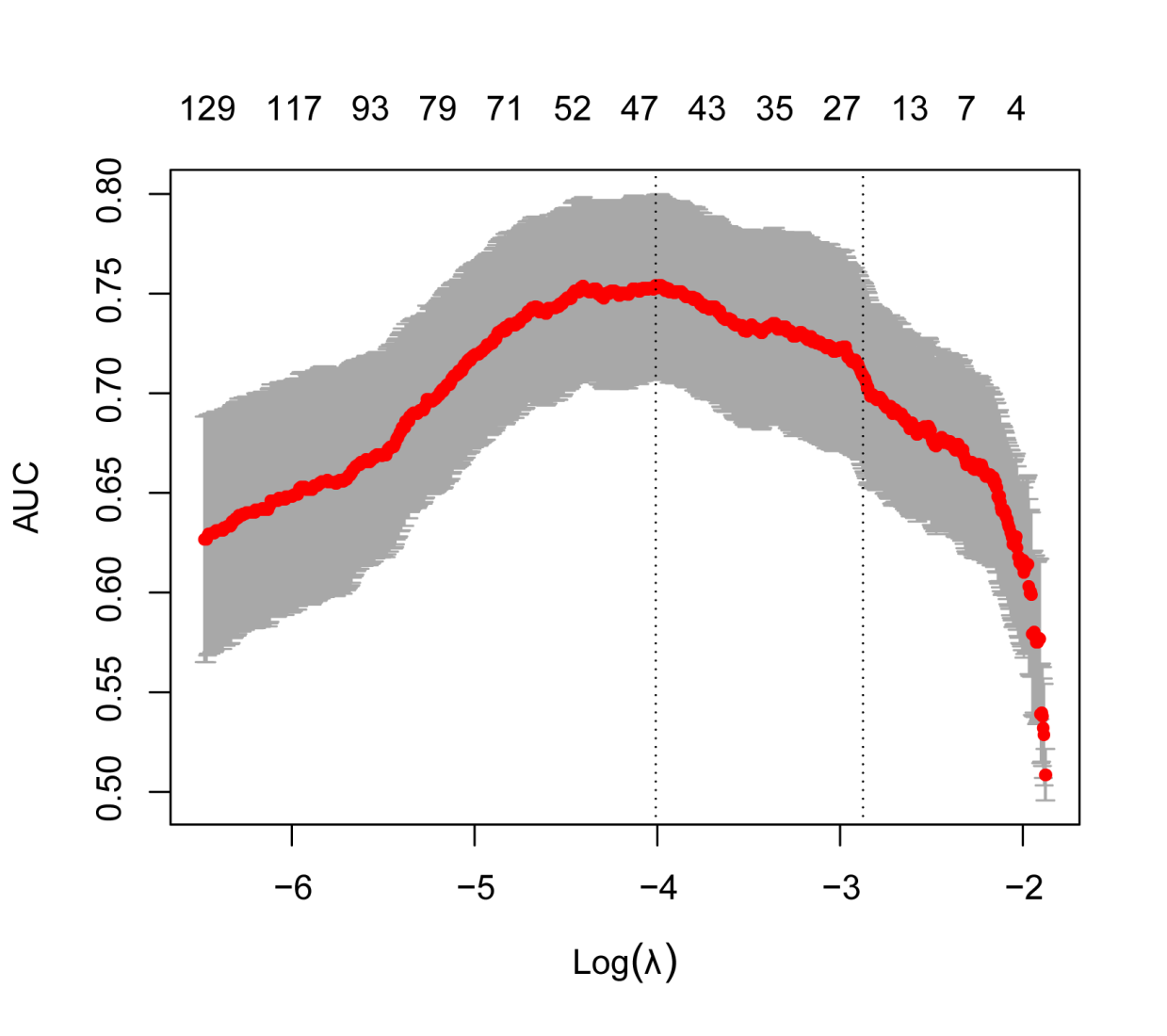


Following LASSO regression, the radiomics features were ultimately compressed to a final set of 47 features.
