# Supplementary material for: B-mode ultrasound and contrast-enhanced ultrasound-based radiomics interpretable analysis for the prediction of macrotrabecular-massive subtype of hepatocellular carcinoma
Source: Ultrasound J. 2025 Oct 17;17:53. doi: 10.1186/s13089-025-00452-2 (PMC12534629; doi:10.1186/s13089-025-00452-2)
Supplement: Supplementary file 4 — Supplementary Material 4. [file 13089_2025_452_MOESM4_ESM.docx]

Table S5. 47 radiomics features

| **Radiomics Feature** | **Coefficient** |
| --- | --- |
| **B mode** | |
| original_firstorder_10Percentile | 0.04648915 |
| original_firstorder_Median | 0.8569729 |
| original_glszm_ZonePercentage | 303.9802 |
| original_glszm_SmallAreaHighGrayLevelEmphasis | 0.04091238 |
| original_ngtdm_Complexity | 0.04552089 |
| original_gldm_DependenceVariance | 27.00983 |
| Ipris_shell0_id_mean | -0.07642144 |
| Ipris_shell2_id_mean | -1.366433 |
| CoLIAGe2D_WindowSize3_Sum Average_firstorder_10Percentile | -2.127699 |
| CoLIAGe2D_WindowSize9_Contrast_firstorder_Median | 1.600022 |
| CoLIAGe2D_WindowSize9_Sum of Squares: Variance_firstorder_Skewness | -72.84827 |
| **Arterial phase** | |
| Normalized_radial_lengths_entropy | -281.5367 |
| Ipris_shell1_id_max | 6.664239 |
| Ipris_shell1_id_mean | 2.37996 |
| Ipris_shell2_id_max | 13.80231 |
| Ipris_shell2_id_mean | 2.604718 |
| Ipris_shell2_id_std | 0.9259708 |
| CoLIAGe2D_WindowSize3_Sum Average_firstorder_Skewness | -123.5724 |
| CoLIAGe2D_WindowSize9_Entropy_firstorder_Variance | 506.7905 |
| wavelet-LHL_lbp-3D-m1_firstorder_RootMeanSquared | -6.338156 |
| wavelet-LHH_lbp-3D-m2_firstorder_Median | -6.201896 |
| wavelet-HLH_lbp-3D-m2_firstorder_90Percentile | -172.8246 |
| **Portal venous phase** | |
| original_firstorder_Maximum | 0.02345738 |
| original_firstorder_Range | 0.4624848 |
| original_glrlm_RunVariance | 0.1149619 |
| original_glszm_GrayLevelVariance | 7.428267 |
| original_glszm_ZoneEntropy | 2.84408 |
| original_ngtdm_Contrast | -1962.94 |
| CoLIAGe2D_WindowSize3_Sum Average_firstorder_Mean | -2.928879 |
| CoLIAGe2D_WindowSize9_Angular Second Moment_firstorder_RobustMeanAbsoluteDeviation | 251.5828 |
| wavelet-LHL_lbp-3D-m1_firstorder_10Percentile | 3.58383E-13 |
| CoLIAGe2D_WindowSize9_Inverse Difference Moment_firstorder_RobustMeanAbsoluteDeviation | 7.96407E-14 |
| CoLIAGe2D_WindowSize9_Sum Average_firstorder_Mean | -2.803914 |
| **Delayed phase** | |
| original_glrlm_RunEntropy | 51.24159 |
| original_glszm_LargeAreaHighGrayLevelEmphasis | 0.0115032 |
| original_gldm_GrayLevelNonUniformity | 0.001403322 |
| Ipris_shell2_id_max | 87.17093 |
| CoLIAGe2D_WindowSize3_Sum Average_firstorder_RootMeanSquared | -22.73538 |
| CoLIAGe2D_WindowSize9_Difference Entropy_firstorder_InterquartileRange | 1.679101 |
| CoLIAGe2D_WindowSize9_Difference Entropy_firstorder_Variance | 754.4712 |
| wavelet-LHL_lbp-3D-m1_firstorder_10Percentile | 195.9129 |
| wavelet-LHL_lbp-3D-m2_firstorder_10Percentile | 14.87044 |
| wavelet-LHH_lbp-3D-m1_firstorder_InterquartileRange | -13.45695 |
| wavelet-HLL_lbp-3D-m2_firstorder_90Percentile | -93.15735 |
| wavelet-HLL_lbp-3D-k_firstorder_10Percentile | -39.57459 |
| wavelet-HLH_lbp-3D-m2_firstorder_90Percentile | -167.7802 |
| wavelet-HHL_lbp-3D-m1_firstorder_Median | 0.2865945 |
