# Supplementary material for: B-mode ultrasound and contrast-enhanced ultrasound-based radiomics interpretable analysis for the prediction of macrotrabecular-massive subtype of hepatocellular carcinoma
Source: Ultrasound J. 2025 Oct 17;17:53. doi: 10.1186/s13089-025-00452-2 (PMC12534629; doi:10.1186/s13089-025-00452-2)
Supplement: Supplementary file 5 — Supplementary Material 5. [file 13089_2025_452_MOESM5_ESM.docx]

Table S1. Number of cases and ultrasound systems in different centers

| Centers | ultrasound systems | Number of cases |
| --- | --- | --- |
| Zhongshan Hospital | Samsung RS80A with CA1-7A transducer (1.0-7.0 MHz)  Acuson Sequoia with 5C1 transducer (2.0-5.0 MHz)  GE LOGIQ E9 with C1-5-D transducer (1.0-5.0 MHz) | 255 |
| Sun Yat-sen University Cancer Center | Acuson Sequoia with 5C1 transducer (2.0-5.0 MHz) | 65 |
| Shanghai Tenth People's Hospital | GE LOGIQ E9 with C1-5-D transducer (1.0-5.0 MHz) | 24 |
