# Supplementary figures and images for: B-mode ultrasound and contrast-enhanced ultrasound-based radiomics interpretable analysis for the prediction of macrotrabecular-massive subtype of hepatocellular carcinoma
Source: Ultrasound J. 2025 Oct 17;17:53. doi: 10.1186/s13089-025-00452-2 (PMC12534629; doi:10.1186/s13089-025-00452-2)

Figure S6. Training curve of BM+CEUSR model


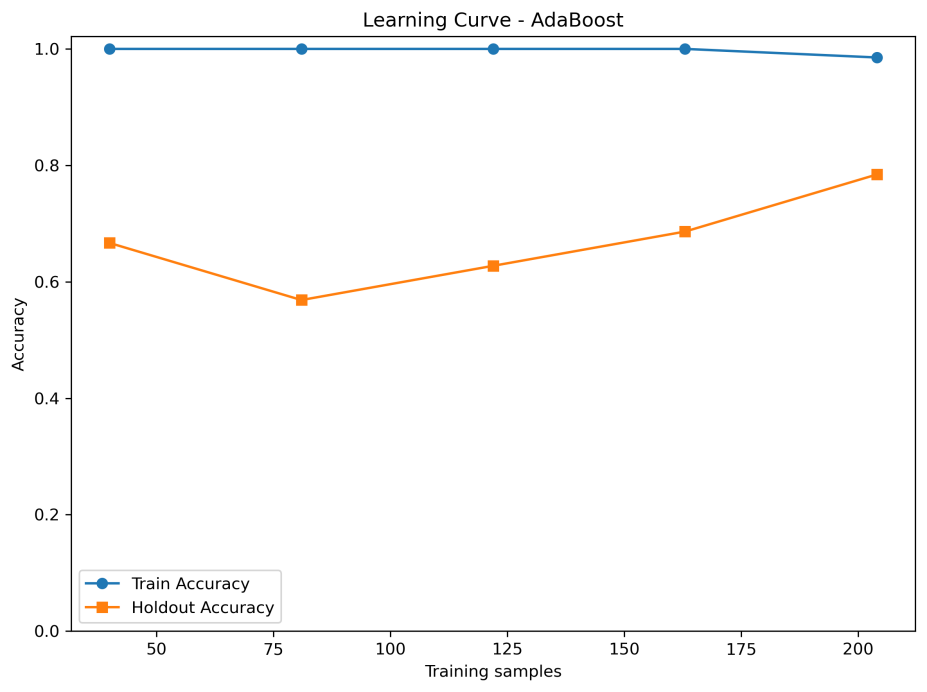

Supplement: Supplementary file 6 — Supplementary Material 6. [file 13089_2025_452_MOESM6_ESM.docx]
