# Supplementary material for: B-mode ultrasound and contrast-enhanced ultrasound-based radiomics interpretable analysis for the prediction of macrotrabecular-massive subtype of hepatocellular carcinoma
Source: Ultrasound J. 2025 Oct 17;17:53. doi: 10.1186/s13089-025-00452-2 (PMC12534629; doi:10.1186/s13089-025-00452-2)

Figure S7. The recurrence-free survival rates in actual non-MTM patients and MTM patients (a) and BM+CEUS_R_ model predicted non-MTM patients and MTM patients (b) in the external centers


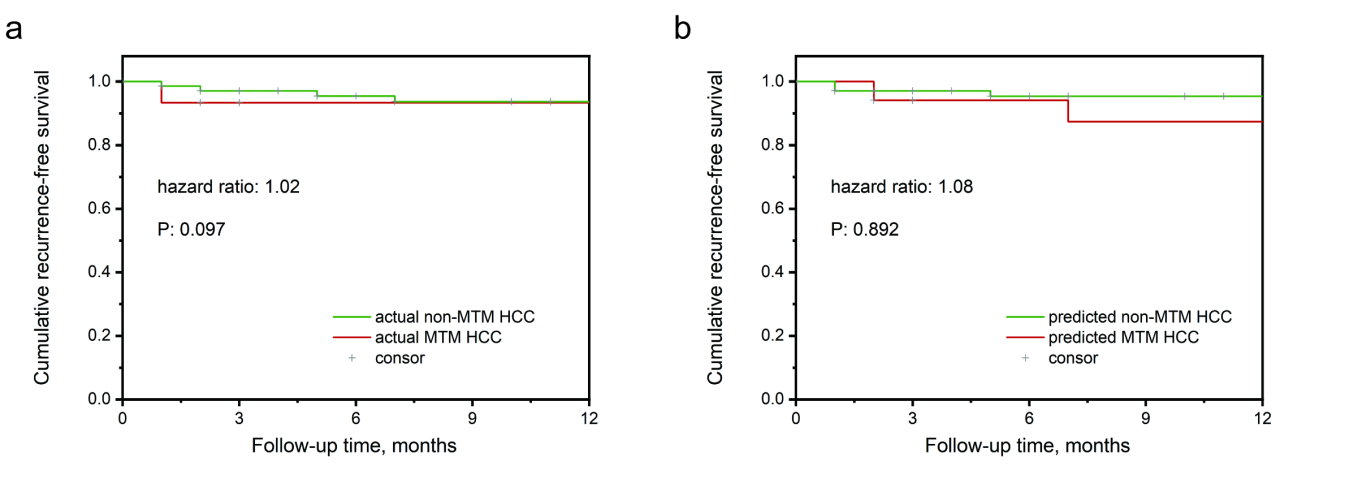

Supplement: Supplementary file 7 — Supplementary Material 7. [file 13089_2025_452_MOESM7_ESM.docx]
